# Clinical profile of recurrent community-acquired pneumonia in children

**DOI:** 10.1186/1471-2466-13-60

**Published:** 2013-10-10

**Authors:** Francesca Patria, Benedetta Longhi, Claudia Tagliabue, Rossana Tenconi, Patrizia Ballista, Giuseppe Ricciardi, Carlotta Galeone, Nicola Principi, Susanna Esposito

**Affiliations:** 1 Department of Pathophysiology and Transplantation, Pediatric Highly Intensive Care Unit, Università degli Studi di Milano, Fondazione IRCCS Ca’ Granda Ospedale Maggiore Policlinico, Via Commenda 9, Milan 20122, Italy; 2 Pediatric Unit Ospedale Bassini Istituti Clinici di Perfezionamento, Cinisello Balsamo (Milan), Italy; 3 Department of Clinical Sciences and Community Health, University of Milan, Milan, Italy

**Keywords:** Allergy, Asthma, Atopy, Children, Community-acquired pneumonia, Lower respiratory tract infection, Pneumonia, Recurrent pneumonia, Respiratory tract infection, Wheezing

## Abstract

**Background:**

The aim of this case–control study was to analyse the clinical characteristics of children with recurrent community-acquired pneumonia (rCAP) affecting different lung areas (DLAs) and compare them with those of children who have never experienced CAP in order to contribute to identifying the best approach to such patients.

**Methods:**

The study involved 146 children with ≥2 episodes of radiographically confirmed CAP in DLA in a single year (or ≥3 episodes in any time frame) with radiographic clearing of densities between occurrences, and 145 age- and gender-matched controls enrolled in Milan, Italy, between January 2009 and December 2012. The demographic and clinical characteristics of the cases and controls were compared, and a comparison was also made between the cases with rCAP (i.e. ≤3 episodes) and those with highly recurrent CAP (hrCAP: i.e. >3 episodes).

**Results:**

Gestational age at birth (p = 0.003), birth weight (p = 0.006), respiratory distress at birth (p < 0.001), and age when starting day care attendance (p < 0.001) were significantly different between the cases and controls, and recurrent infectious wheezing (p < 0.001), chronic rhinosinusitis with post-nasal drip (p < 0.001), recurrent upper respiratory tract infections (p < 0.001), atopy/allergy (p < 0.001) and asthma (p < 0.001) were significantly more frequent. Significant risk factors for hrCAP were gastroesophageal reflux disease (GERD; p = 0.04), a history of atopy and/or allergy (p = 0.005), and a diagnosis of asthma (p = 0.0001) or middle lobe syndrome (p = 0.001). Multivariate logistic regression analysis, adjusted for age and gender, showed that all of the risk factors other than GERD and wheezing were associated with hrCAP.

**Conclusions:**

The diagnostic approach to children with rCAP in DLAs is relatively easy in the developed world, where the severe chronic underlying diseases favouring rCAP are usually identified early, and patients with chronic underlying disease are diagnosed before the occurrence of rCAP in DLAs. When rCAP in DLAs does occur, an evaluation of the patients’ history and clinical findings make it possible to limit diagnostic investigations.

## Background

Recurrent community acquired pneumonia (rCAP) is not rare in children living in industrialised countries. The Isle of Wight birth cohort study of 1,336 children followed up for 10 years found a 7.4% prevalence of ≥2 lower respiratory tract infections (LRTIs) including CAP [1]; a retrospective cohort study of German children aged 5–7 years found that 6.7-8.2% of the children had a positive history of CAP, 6.9-8.2% of whom had experienced rCAP [2]; and data from Toronto’s Hospital for Sick Children show that 238 of 2,900 children (8%) admitted because of CAP met the criteria for rCAP [3].

Identifying the factors that can favour the development of a new CAP episode is critical for the implementation of appropriate preventive, diagnostic and therapeutic measures. However, although it is relatively easy to evaluate and treat cases occurring in the same lung region, it is more difficult in the case of rCAP developing in different lung areas (DLAs). Recurring lung densities in the same regions are almost invariably due to intra- or extraluminal bronchial obstruction, or structural abnormalities of the airways or lung parenchyma, and the recommended airway endoscopy and/or diagnostic imaging (sometimes in association with laboratory tests for tuberculosis and fungal diseases) is almost always sufficient to make a diagnosis [4]. On the contrary, rCAP affecting multiple lobes or different areas of the same lobe may be associated with a wide range of more or less severe clinical problems that could *per se* increase the risk of lung infection. As some of these can only be identified using specific laboratory and/or instrumental methods, diagnosing and treating patients with rCAP in DLAs can be particularly complicated and expensive, and is not always efficacious.

Various attempts have been made to define the most frequent causes of rCAP and establish the most rational diagnostic and therapeutic approach [5-9]. Unfortunately, the findings are frequently conflicting, and it is not possible to state whether the cause of rCAP in DLAs is necessarily a serious underlying diseases requiring immediate diagnosis.

The aim of this case–control study was to analyse the demographic and clinical characteristics as well as the predisposing factors of children with rCAP in DLAs and compare them with those of children who had never experienced CAP in order to contribute to identifying the best approach to such patients.

### Patients and methods

#### Patients

We reviewed the medical records of all of the children aged <14 years who attended the Respiratory Disease Section of Pediatric Clinic 1, Department of Pathophysiology and Transplantation, University of Milan, Fondazione IRCCS Ca’ Granda, Ospedale Maggiore Policlinico, Milan, Italy, between January 2009 and December 2012 because of rCAP. The patients were included in the analysis if they had experienced ≥2 episodes of radiographically confirmed CAP in a single year (or ≥3 episodes in any time frame) with radiographic clearing of densities between occurrences [10]. Only the children with rCAP in DLAs were enrolled, except for those with a diagnosis of cystic fibrosis identified in the first weeks of life who were excluded and followed up in accordance with specific diagnostic and therapeutic guidelines. The controls were age- and gender-matched children who had never experienced CAP, and for whom all of the data concerning their health problems since birth were available as they had been continuously followed at the same institution for the purpose of vaccinations and periodic evaluations of growth and psychological development.

The study was approved by the Ethics Committee of Fondazione IRCCS Ca’ Granda. Ospedale Maggiore Policlinico, Milan, Italy, and written informed consent was obtained from the parents or legal guardian of all of the enrolled children.

## Methods

A previously prepared electronic chart was used to record each enrolled child’s age, gender, height, weight, gestational age, birth weight, respiratory complications at birth, duration of breast-feeding, the number of siblings, type of dwelling (i.e. urban or rural), household exposure to cigarette smoke, age when starting day care attendance, and the number and type of CAP episodes. We also recorded any underlying diseases possibly associated with CAP that had been diagnosed during hospitalisation or a visit to our outpatient clinic: i.e. oromotor incoordination and swallowing dysfunction predisposing to aspiration syndrome [11]; immune disorders, including atopy/allergy and primary and acquired immunodeficiency syndrome [12,13]; congenital heart defects [14]; lung and airway problems such as chronic rhinosinusitis with post-nasal drip [15], primary ciliary dyskinesia [16], recurrent wheezing [17,18], bronchial asthma [13] or middle lobe syndrome [19]; gastroesophageal reflux disease (GERD) [20]; and overweight or obesity [21].

Aspiration syndrome was diagnosed clinically and confirmed by means of fluoroscopic feeding studies showing oropharyngeal incoordination. Immune function was determined by measuring serum immunoglobulin levels, IgG subclasses, T cell subpopulations, and complement assays. Atopy was diagnosed in the presence of total IgE levels of ≥2 standard deviations (SDs) for age and/or allergen-specific IgE for common inhaled and food allergens. The diagnoses of allergy were based on clinical symptoms, skin tests and IgE determinations. Cardiac anomalies were confirmed by means of echocardiography. Chronic rhinosinusitis with post-nasal drip was clinically diagnosed in the presence of at least two of the following symptoms: a mucous or purulent nasal discharge for more than three months, nasal congestion, and posterior nasal drainage; in the case of doubt or the frequent recurrence of pulmonary infections, flexible optical fibre nasal endoscopy and/or paranasal sinus computed tomography were used to confirm the diagnosis and identify any mechanical obstructions of the upper airways. Nasal ciliary function was evaluated by measuring ciliary beat frequency in samples of ciliated epithelium from the inferior nasal turbinate; in patients with two pathological values, a further brushing was taken for ultrastructural analysis by means of electron microscopy. A diagnosis of suspected GERD was based on clinical history and the assessment of symptoms such as regurgitation, nausea, chest and/or abdominal pain, hoarseness and dysphagia; when the diagnosis was doubtful, or in the presence of refractory respiratory or gastrointestinal manifestations, the children underwent a 24-hour intra-esophageal pH study. The percentage of body fat was calculated as the body mass index (BMI) SD in accordance with the World Health Organisation child growth standards. Recurrent wheezing in pre-school children was defined as at least three episodes of respiratory tract infections in a period of six months with parental reports of shortness of breath and physician-diagnosed dyspnea [17,18]. Bronchial asthma in school-age children was diagnosed in the presence of at least two episodes of wheezing, breathlessness, chest tightness, coughing, and pulmonary function test evidence of airflow limitation. Right middle lobe syndrome was defined as atelectasis persisting for more than 4–6 weeks or two or more episodes of consolidation of the right middle lobe.

### Statistical analysis

The continuous variables are given as mean values ± SD and were compared between groups using a two-sided Student’s *t* test after checking that the data were normally distributed; and the categorical variables are given as numbers and percentages and were compared using contingency table analysis and the chi-squared or Fisher’s test, as appropriate.

The odds ratios (ORs) of rCAP and corresponding 95% confidence intervals (CIs) were computed using conditional logistic regression, stratified by gender and age.

In further analyses, comparisons were made between the children with rCAP (i.e. ≤3 episodes) and those with hrCAP (i.e. >3 episodes). The hrCAP/rCAP ORs and CIs were computed using unconditional multiple logistic regression models, including terms for gender and age (<7 *vs* ≥7 years old: i.e. median values).

All of the tests were two-sided and a p value of <0.05 was considered statistically significant. The data were analysed using SAS, version 9.1 (Cary, NC, USA).

## Results and discussion

Table 1 shows the demographic characteristics of the 146 children with rCAP in DLAs and 145 controls enrolled in the study. The two groups were comparable in terms of gender and age distribution, the duration of breast-feeding, the type of dwelling, exposure to cigarette smoke and the number of siblings, but gestational age at birth (p = 0.003), birth weight (p = 0.006), respiratory distress at birth (p < 0.001) and age when starting day care attendance (p = 0.001) were significantly different.

**Table 1:** Demographic characteristics of the study population

	**Patients with rCAP**	**%**	**Healthy controls**	**%**	**Comparison between groups**
	**(n = 146)**		**(n = 145)**		p*
Gender					
Female	73	50.0	73	50.0	0.95
Male	73	50.0	72	50.0	
Age, mean ± SD (yrs)	7.9 ± 4.5		8.1 ± 4.5		0.70
Gestational age (weeks)					
<32	11	7.5	0	0	
32-36	18	12.3	17	11.7	0.003
≥37	117	80.2	128	88.3	
Birth weight (g)					
<750	3	2.1	0	0	
750-1,000	1	0.7	0	0	
1,001-1,500	7	4.8	0	0	0.006
1,501-2,500	14	9.6	7	4.8	
>2500	121	82.8	138	95.2	
Respiratory distress at birth					
Yes	26	17.8	0	0	<0.001
No	120	82.2	145	100	
Breast-feeding >3 months					
Yes	83	58.4	85	58.6	0.94
No	59	41.6	60	41.4	
Type of dwelling					
Urban	127	87.0	124	85.5	0.72
Rural	19	13.0	21	14.5	
Exposure to cigarette smoke					
Yes	55	37.7	45	31.0	0.23
No	91	62.3	100	69.0	
Number of siblings					
0	48	32.9	45	31.0	0.08
1	66	45.2	74	51.0	
2	26	17.8	26	17.9	
≥3	6	4.1	0	0	
Age starting day care attendance					
≤1 year	14	9.6	0	0	<0.001
>13-23 months	24	16.4	2	1.4	
24-35 months	33	22.6	40	27.6	
36-47 months	64	43.8	103	71.0	
≥4 years	11	7.5	0	0	

*rCAP*: recurrent community-acquired pneumonia; *SD*: standard deviation. *P values were calculated using Student’s *t* test, the chi-squared test or Fisher’s test, as appropriate.

Table 2 shows the prevalence of possible predisposing factors in the two groups. Severe predisposing factors such as heart disease, oromotor incoordination and swallowing dysfunction, ciliary dyskinesia, and primary or acquired immunodeficiency were rare and never observed in the controls, but were diagnosed before rCAP in DLAs in the cases. Middle lobe syndrome was quite frequent among the cases but never reported among controls. GERD and obesity were relatively common in both groups, but some conditions involving the respiratory tract were more frequent in the children with rCAP. Recurrent infectious wheezing was diagnosed in 113 cases (77.4%) and in only 20 controls (13.8%)(p < 0.001); chronic rhinosinusitis with post-nasal drip was diagnosed in respectively 105 (71.9%) and six (4.1%) (p < 0.001); recurrent upper respiratory tract infections in 99 (67.8%) and 29 (20.0%) (p < 0.001); atopy/allergy in 75 (51.4%) and 17 (11.7%; p < 0.001); and asthma in 46 (31.5%) and eight (5.5%)(p < 0.001). The most important predisposing factors were rhinosinusitis, recurrent wheezing and asthma, which respectively increased the risk of rCAP by about 60, 32 and 10 times.

**Table 2:** **Predisposing factors associated with CAP recurrences in children with rCAP and controls**, **and corresponding odds ratios** (**ORs**) **with 95% confidence intervals** (**95% CI**)

	**Patients with rCAP**		**Healthy controls**		**Comparison between groups**	**OR (95% CI)**^**§**^
	(n = 146)	%	(n = 145)	%	*p*°°	
***Predisposing factor***						
Heart disease						
Yes	3	2.1	0	0	0.25	-
No	143	97.9	145	100.0		
Oro-motor incoordination/swal-lowing dysfunction						
Yes	4	2.7	0	0	0.12	-
No	142	97.3	145	100.0		
Primary ciliary dyskinesia						
Yes	1	0.7	0	0	1.00	-
No	145	99.3	145	100.0		
Primary or acquired immunodeficiency						
Yes	2	1.4	0	0	0.50	-
No	144	98.6	145	100.0		
GERD						
Yes	31	21.2	20	13.8	0.69	1.33 (0.64-2.80)
No	115	78.8	125	86.2		1^§§^
Overweight/obesity						
BMI SD <1	112	76.7	119	82.1	0.76	1^§§^
BMI SD ≥1	34	23.3	26	17.9		1.34 (0.58-1.97)
Wheezing						
Yes	113	77.4	20	13.8	<0.001	32.00 (10.14-100.96)
No	33	22.6	125	86.2		1^§§^
Chronic rhinosinusitis with post-nasal drip						
Yes	105	71.9	6	4.1	<0.001	59.33 (24.48-144.97)
No	41	28.1	139	95.9		1^§§^
Middle lobe syndrome						
Yes	44	30.1	0	0		
No	102	69.9	145	100.0	<0.001	-
Recurrent upper respiratory tract infections						
Yes	99	67.8	29	20.0	<0.001	6.75 (3.68-12.37)
No	47	32.2	116	80.0		1^§§^
Atopy/allergy						
Yes	75	51.4	17	11.7	<0.001	7.44 (3.71-14.93)
No	71	48.6	128	88.3		1^§§^
Asthma						
Yes	46	31.5	8	5.5	<0.001	10.50 (3.77-29.28)
No	100	68.5	137	94.5		1^§§^

*CI*: confidence interval; *BMI SD*: body mass index standard deviation; *GERD*: gastroesophageal reflux diseae; *OR*: odds ratio. °°Chi-squared or Fisher’s test, as appropriate; *rCAP*: recurrent community-acquired pneumonia. ^§^ORs from conditional logistic regression model, stratified by gender and age.^§§^reference category.

Table 3 compares the patients with rCAP (≤3 episodes) and those with hrCAP (>3 episodes), and shows that the significant risk factors for hrCAP were GERD (p = 0.04), a history of atopy and/or allergy (p = 0.005), a diagnosis of asthma (p = 0.0001), and middle lobe syndrome (p = 0.001); the last three were confirmed in the multivariate logistic regression analysis adjusted for age and gender, which also indicated that wheezing was associated with hrCAP.

**Table 3:** **Comparison of predisposing factors between children with rCAP** (≤**3**) **and those with hrCAP** (>**3 CAP**)

**Predisposing factor**	**≤3 CAP**		**>3 CAP**		**Comparison between groups**	**OR (95% CI)**^**§**^
	**Total (n = 71)**	**%**	**Total (n = 75)**	**%**	***p°***	
GERD						
Yes	10	14.1	21	28.0	0.04	2.34 (0.07-5.69)
No	61	85.9	54	72.0		1^§§^
Overweight/obesity						
BMI SD <1	58	81.7	54	72.0	0.53	1^§§^
BMI SD ≥1	13	18.3	21	28.0		2.18 (0.53-8.91)
Wheezing						
Yes	51	71.8	62	82.7	0.056	2.43 (1.02-5.79)
No	20	28.2	13	17.3		1^§§^
Chronic rhinosinusitis with post nasal drip						
Yes	46	64.8	59	78.7	0.06	1.97 (0.91-4.27)
No	25	35.2	16	21.3		1^§§^
Middle lobe syndrome						
Yes	12	16.9	32	42.7		3.02 (1.36-6.71)
No	59	83.1	43	57.3	<0.001	1^§§^
Recurrent upper respiratory tract infections						
Yes	47	66.2	52	69.3	0.69	1.33 (0.64-2.80)
No	24	33.8	23	30.7		1^§§^
Atopy/allergy						
Yes	28	39.4	47	62.7	0.005	2.20 (1.10-4.42)
No	43	60.6	28	37.3		1^§§^
Asthma						
Yes	11	15.5	35	46.7	<0.001	3.46 (1.48-8.08)
No	60	84.5	40	53.3		1^§§^

*CI*: confidence interval; *BMI SD*: body mass index standard deviation; *GERD*: gastroesophageal reflux disease; *OR*: odds ratio; °Chi-squared test; ^§^estimates from multiple logistic regression models, including terms for gender and age (<7 *vs* ≥7 years old, median values); ^§§^reference category.

To the best of our knowledge, this is the first study carried out in an industrialised country evaluating the prevalence of factors that are thought to be associated with an increased risk of developing rCAP in DLAs in children with or without rCAP. As some of the factors associated with an increased risk of rCAP in DLAs are very common pediatric diseases, this approach should better define the real importance of each and simultaneously quantify the real weight of other potentially negative predisposing factors.

The study data suggest that children living in an industrialised country with a history of rCAP in DLAs can be divided into those suffering from a severe underlying disease that favours the development of CAP but also causes other significant signs and symptoms that frequently involve extra-respiratory sites, and those whose clinical history is no different from that of many children without rCAP. Some generally mild diseases were frequent in both groups, although slightly more among the cases; severe underlying diseases were fortunately very rare and had already been identified before they could cause rCAP. Heart disease, oromotor incoordination and swallowing dysfunction predisposing to aspiration syndrome, primary or acquired immunodeficiency syndrome, and primary ciliary dyskinesia were observed in very few cases, which indicates that the children had been adequately diagnosed and treated from their first months of life, thus significantly reducing the risk of multiple CAP episodes. The same can probably also be said of cystic fibrosis because neonatal screening in Milan allows the early identification of the vast majority of cases so that these patients were excluded. However, our findings are different from those of most other studies of the potential causes of rCAP [3,7,8]. It has been reported that severe underlying diseases are not uncommon, may be unknown when a child with rCAP is examined for the first time and, consequently, may justify a more aggressive diagnostic approach in a large number of subjects. Owayed *et al*. examined the clinical records of 238 children with a history meeting the criteria for rCAP, and found that 48% of them had aspiration syndrome, 10% immune disorders and 9% congenital heart disease [3]. Adam found that about half of the children with rCAP that he studied had immunodeficiency or immotile cilia syndrome [22]. Another retrospective study investigating the cause of rCAP in 70 Indian children found that the most frequent illness was recurrent aspiration, followed by immunodeficiency [7], and a more recent Spanish study found that congenital cardiac defects and aspiration syndrome were the most represented illnesses [8]. However, most of these studies were carried out some years ago and some were conducted in the developing world, where there is still a lack of basic services, high morbidity and mortality rates, and where the identification of severe underlying diseases leading to rCAP is regularly delayed.

Unlike severe diseases, mild to moderate respiratory infections were significantly more frequent in our children with rCAP, which seemed to be significantly associated with wheezing, chronic rhinosinusitis with postnasal drip, atopy/allergy and asthma. Recurrent respiratory tract infections are common in otherwise healthy children during the first years of life [23] because of the relative immaturity of the immune system, and they can lead to more severe disease in atopic and allergic children, and those with wheezing or asthma [23-25]. Most of our patients with rCAP in DLAs were simultaneously affected by all of these conditions, which suggests that the concurrence of several factors simply increased the clinical evidence of a natural phenomenon but had no particular clinical implication. Consequently, such children only need to undergo the preventive and therapeutic measures usually prescribed for recurrent respiratory infections and wheezing.

Although chronic rhinosinusitis with post-nasal drip is a common childhood disease [26,27], it is not usual to examine the upper airways and seek a purulent discharge inside the nasal cavities in children with a history of rCAP. However, on the basis of our results, it is important to start thinking in terms of nose-lung interactions as in the case of the relationship between allergic rhinitis and asthma [28]. The relevance of asthma as a possible cause of rCAP has been reported by a number of authors, and seems to be strictly related to the quality of asthma control [17,28,29]. The lung function of children whose asthma is well controlled by inhaled corticosteroids is normal, and they experience very few (if any) lower respiratory tract infections. Consequently, they do not need a particularly aggressive diagnostic approach and should simply be prescribed adequate asthma prophylactic therapy. A limitation of our study is represented by the absence of etiologic data on CAP episodes reported in the study population. It is possible that a proportion of children presenting with CAP have had a viral rather than bacterial cause of rCAP. Viral infections have been frequently associated with atopy, asthma and allergy [30,31] and these viral infections could explain our findings. However, also CAP due to atypical as typical bacteria can be associated with asthma [18,32,33].

In the case of right middle lobe syndrome associated with hrCAP, our data support the prompt use of physiotherapy with a positive pressure mask in order to aid regional lung clearance and avoid CAP recurrences [34]. Some authors have found a relationship between right middle lobe syndrome and asthma [34,35] but the small number of patients in our series prevents us from confirming or refuting this finding.

We did not find that GERD increased the risk of rCAP, although it may be associated with more frequent recurrences. This is not in line with the findings of some uncontrolled studies [3,7,9] but as GERD is a common disease, it is possible that the observed relationship was only casual. In any case, GERD can be easily diagnosed on the basis of gastrointestinal complaints and treated without any further laboratory or radiological investigations.

Our findings indicate that prematurity, a low birth weight and respiratory distress at birth are independent factors associated with rCAP. The widely reported importance of chronic respiratory morbidity in prematurely born infants is due to their smaller and hyper-responsive airways [36-38], and there is also evidence that they may also have a genetic predisposition to viral infections [39], a combination that can facilitate catarrhal obstruction of the airways and lung inflammation. Up to 73% of pre-term infants with pulmonary bronchodysplasia require readmission to hospital because of lower respiratory tract diseases in the first years of life, and chronic respiratory morbidity requiring the use of healthcare facilities remains even at school age [40]. It should be investigated whether, in addition to adequate vaccinations, all pre-term babies and those with respiratory distress at birth would benefit from regular chest physiotherapy during respiratory illnesses in order to enhance secretion mobilisation and prevent recurrent infections. Moreover, as most of our children with rCAP were allergic and asthmatic, our finding that early day care attendance seemed to protect against rCAP supports the idea that the so-called "hygiene hypothesis" (i.e. exposure to a wide variety of pathogens protects against atopy and asthma) might also apply to rCAP [41].

## Conclusions

In brief, our findings show that the diagnostic approach to children with rCAP in DLAs used in the developed world is relatively easy in most cases, and the severe underlying diseases favouring rCAP are usually identified early. Furthermore, when episodes of rCAP in DLAs do occur, an adequate evaluation of the patient’s history and clinical findings is usually sufficient. Except for prematurity, the factors predisposing children to develop rCAP in DLAs are of limited clinical importance and do not need any particular diagnostic measures. Wheezing, chronic rhinosinusitis with postnasal drip, atopy/allergy and asthma seem to be the main factors associated with CAP recurrences and, in their presence, children with rCAP can be followed up by means of the same procedures as those usually used in the case of children without rCAP. Our findings have relevance in terms of informing future research on potential risk factors for recurrent CAP, although a more complicated approach is needed in the third world, where the early identification of the severe underlying diseases favouring rCAP is significantly more difficult or even impossible.

## Abbreviations

BMI: Body mass index; DLA: Different lung areas; GERD: Gastroesophageal reflux disease; OR: Odds ratio; rCAP: recurrent community-acquired pneumonia; SD: Standard deviation.

## Competing interests

The authors declare that they have no competing interests.

## Authors’ contributions

MFP drafted the manuscript, and was head of the outpatient clinic at which the patients with rCAP were enrolled; BL, CT, RT, PB and GR participated in the enrolment of the rCAP patients and otherwise healthy subjects; CG made the statistical analysis; NP revised the draft of the manuscript and made a substantial scientific contribution; SE coordinated and supervised the enrolment of the rCAP patients and otherwise healthy subjects, and co-wrote the draft manuscript. All of the authors read and approved the final version of the manuscript.

## Pre-publication history

The pre-publication history for this paper can be accessed here:

http://www.biomedcentral.com/1471-2466/13/60/prepub
